# Platelet Count Might Be Associated With the Closure of Hemodynamically Significant Patent Ductus Arteriosus

**DOI:** 10.3389/fped.2021.729461

**Published:** 2021-10-11

**Authors:** Junyan Zhong, Binchun Lin, Yongping Fu, Yanliang Yu, Jie Zhao, Depeng Zhao, Chuanzhong Yang, Xueyu Chen

**Affiliations:** ^1^Department of Neonatology, Shenzhen Maternity and Child Healthcare Hospital, The First School of Clinical Medicine, Southern Medical University, Shenzhen, China; ^2^Department of Reproductive Medicine, Shenzhen Maternity and Child Healthcare Hospital, The First School of Clinical Medicine, Southern Medical University, Shenzhen, China

**Keywords:** ibuprofen, patent ductus arteriosus, platelets, ductal closure, pharmaceutical treatment

## Abstract

**Background:** Platelet-rich thrombosis leads to the occlusion of arteries. Whether the association between platelet count and closure of hemodynamically significant patent ductus arteriosus (hsPDA) exists remains inconclusive. Given that neonatal platelet count is significantly affected by infection, this study aims to evaluate the association of platelet parameters before ibuprofen treatment with the closure of hsPDA in very low birth weight (VLBW) infants without concurrent infection.

**Methods:** A retrospective study was conducted at the NICU of Shenzhen Maternity and Child Healthcare Hospital from January 2016 to August 2020. VLBW infants diagnosed with hsPDA, treated with oral ibuprofen and without concurrent infection were included in this study. The platelet parameters were retrieved from the whole-blood test routinely performed within 24 h before starting treatment of oral ibuprofen. A multiple regression model was built to evaluate the association between platelet parameters before ibuprofen treatment and successful closure of hsPDA.

**Results:** A total of 129 premature infants with hsPDA were analyzed in this study. After oral ibuprofen treatment, successful closure of hsPDA was achieved in 70 (54.3%) infants. The gestational age at birth and birth weight in infants with successful or failed closure of hsPDA after ibuprofen treatment were 28.3 vs. 27.6 weeks (*p* = 0.016) and 1,120 vs. 960 g (*p* = 0.043), respectively. The rate of mechanical ventilation in infants with successful closure of hsPDA was significantly lower compared to those with failed closure of hsPDA, 31.4 vs. 54.2%, *p* = 0.014. The platelet count in infants with successful closure of hsPDA after ibuprofen treatment was significantly higher compared to those with failed closure of hsPDA, 212 vs. 183 (in a unit of 10^9^/L), respectively (*p* = 0.024). Multivariate logistic regression analysis showed that a higher platelet count (≥181 × 10^9^/L) before ibuprofen treatment was independently associated with successful closure of hsPDA [odds ratio 2.556, 95% confidence interval (1.101–5.932), *p* = 0.029].

**Conclusion:** The findings in this study suggest that a higher platelet count before oral ibuprofen treatment may predict the probability of successful closure of hsPDA in VLBW infants.

## Introduction

Fetal ductus arteriosus is the vascular channel between the systemic circulation and the pulmonary circulation, supporting the pulmonary circulation during intrauterine life. In term infants, ductus arteriosus normally closes within 10–15 h after birth. However, the persistence of a patent ductus arteriosus (PDA) occurs in more than 30% of premature infants (1). A small PDA causes mild clinical symptoms, while a hemodynamically significant PDA (hsPDA) diagnosed by echocardiography may lead to substantial neonatal morbidities like pulmonary edema and systemic hypoperfusion (2). Despite that there is no census whether to close PDA (3), cyclooxygenase (COX) inhibitors are wildly used for the treatment of hsPDA. Oral ibuprofen is reported to be the most effective COX to close hsPDA (4). A closure of hsPDA was achieved *via* contraction of smooth muscle and formation of platelet-rich thrombosis regulated by many factors, including oxygen sensing system, glutamate, osmolality, prostaglandin E_2_, nitric oxide, and carbon monoxide (1, 5). Recently, studies have found that platelets were also involved in hsPDA closure. Echtler et al. found that platelets were recruited to the luminal side of DA during its closure, and they also confirmed thrombocytopenia or low platelet was independently associated with failure of hsPDA closure (6). However, later studies reported controversial findings regarding the effect of platelet level or function on PDA and its closure (7–9). In addition, whether the platelet level affects the closure of hsPDA by COX inhibitors is also under dispute (7, 9–11).

Although platelets are key to the formation of thrombosis occulting arteries, this process is subject to various mediators such as inflammation and infection. Multiple studies show that inflammation and infection regulate the quantity and function of platelets (12). In inflammatory response, activated vascular endothelia release Weibel-Palade bodies containing von Willebrand factor, which promotes platelet recruitment (13). As a result, the conflicting findings regarding the role of platelets in the closure of hsPDA may be attributed to the heterogeneous population of premature infants complicated with different morbidities like inflammation and infection. Moreover, infection *per se* can result in late ductal reopening and PDA closure failures (14). To minimize the influence of infection on the association between platelets and hsPDA closure, we included hsPDA infants without concurrent infection. This study aims to assess the association between platelet parameters and the closure of hsPDA by ibuprofen.

## Materials and Methods

### Patients and Data Collection

This retrospective study was performed at the NICU of Affiliated Shenzhen Maternity and Child Healthcare Hospital, Southern Medical University, from January 2016 to August 2020. Premature infants were included if (1) born with a birth weight <1,500 g, (2) diagnosed with hsPDA, and (3) received ibuprofen treatment. HsPDA was diagnosed when the ductus diameter exceeds 1.5 mm, left atrial inner diameter/aortic root (LA/AO) exceeds 1.4, and combined left to right shunt by echocardiography (2). We excluded infants from pregnancies with maternal complications such as preeclampsia, maternal autoimmune diseases, and intrauterine infection. Infants with congenital heart diseases, fetal and neonatal alloimmune disorders, intrauterine growth restriction (IUGR), and concurrent infection (defined as proven or suspected early and late onset sepsis, meningitis, pneumonia, and other infections with clinical symptoms such as fever, abnormal white blood cell count, elevated C-reactive protein within 7 days before or after the Ibuprofen treatment) were also excluded from this study. Finally, infants with ibuprofen treatment started later than 7 days of postnatal age due to fasting were also excluded.

Infants' characteristics were collected, including gestational age (GA), birth weight (BW), gender, mode of delivery, antenatal glucocorticoid use, twins, APGAR scores at 1 and 5 min, and mechanical ventilation (≥24 h). Platelet counts (PLT), plateletcrit (PCT), mean platelet volume (MPV), and platelet distribution width (PDW) were retrieved from a whole blood test routinely performed within 24 h before ibuprofen treatment using the Mindray 5390 analyzer platform (Shenzhen, China). The standard biosecurity and institutional safety procedures were not relevant for the current study.

All preterm infants received echocardiography within 5 days after birth or when clinically indicated. Infants with hsPDA received oral ibuprofen (Shanghai Johnson Pharmaceutical Co., Ltd^®^ Specification: 15 ml: 0.6 g) at a daily dose of 10 mg/(kg·day) for 3 consecutive days (4, 15). Successful PDA closure was defined as the absence of PDA shunt confirmed by echocardiography 24–72 h after the treatment.

### Statistics

Student *t*-test or Mann–Whitney *U*-test was used for comparison of continuous variables in independent samples, as appropriate. Chi-square or Fisher's exact tests were used to analyze categorical variables. The data were presented as mean (standard deviation) or median [interquartile range (IQR)] for continuous variables and frequency (percentage) for categorical variables. Receiver operating characteristic (ROC) curve was used to determine the cutoff for platelet parameters. Multivariable logistic regression was used to assess the independent contribution of potential factors to the outcome. The odds ratio and 95% confidence interval were calculated. Statistical analysis was performed with the IBM SPSS Statistics 24 software. A *p* < 0.05 was considered statistically significant.

## Results

### Patients' Characteristics

**Figure 1:**
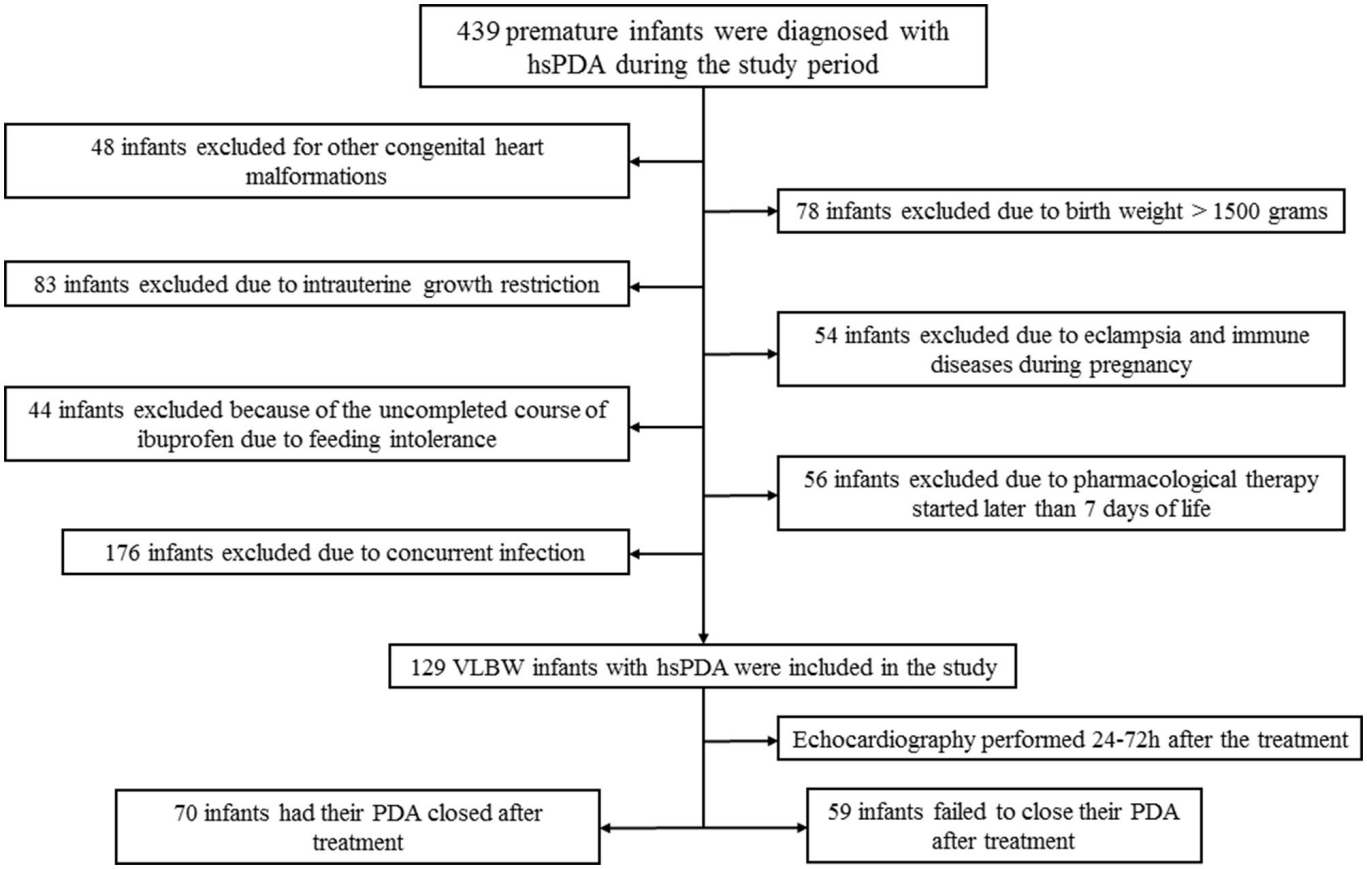
Flow chart of cases selection. hsPDA, hemodynamically significant patent ductus arteriosus; GA, gestational age.

A total of 439 premature infants were diagnosed with hsPDA during the study period. After applying the inclusion and exclusion criteria, 129 infants were analyzed in this study (shown in Figure 1). The demographical and clinical characteristics of these 129 infants are shown in Table 1. Successful closure of hsPDA after the first course of oral ibuprofen treatment was achieved in 70 (54.3%) infants. The gestational age at birth and birth weight in infants with successful or failed closure of hsPDA after ibuprofen treatment were 28.3 vs. 27.6 weeks (*p* = 0.016) and 1,120 vs. 960 g (*p* = 0.043), respectively. The rate of mechanical ventilation in infants with successful closure of hsPDA was significantly lower compared to those with failed closure of hsPDA, 31.4 vs. 54.2%, *p* = 0.014 (Table 1). Univariate analysis showed that the platelet count before the first course in infants with successful closure of hsPDA after ibuprofen treatment was significantly higher compared to those with failed closure of hsPDA, 212 vs. 183 × 10^9^/L (*p* = 0.024, Table 2). The rate of thrombocytopenia before the first course of ibuprofen treatment was significantly higher in infants with failed hsPDA closure compared to those with successful hsPDA closure (33.9 vs. 17.1%, *p* = 0.028, Table 2). Other platelet parameters during the first and second course of treatment were not significantly different between the two groups (Table 2).

**Table 1:** Demographical and clinical characteristics of successful and failed PDA closure after Ibuprofen treatment.

**Variates**	**PDA closure (*N* = 70)**	**PDA open (*N* = 59)**	***p***
Male, *n* (%)	26 (37.1%)	29 (49.2%)	0.169
Gestational age (weeks)	28.3 (27.2–29.5)	27.6 (26.1–28.4)	0.016
Birth weight (g)	1,120 (940–1,265)	960 (840–1,230)	0.043
Twins (*n*, %)	17 (24.3%)	24 (40.7%)	0.062
Vaginal delivery	29 (41.4%)	30 (50.8%)	0.365
Apgar at 1 min	8 (6–10)	8 (5–10)	0.217
Apgar at 5 min	10 (9–10)	10 (9–10)	0.952
MV (>24 hour)	22 (31.4%)	32 (54.2%)	0.014
Time of starting treatment (days)	4 (3–6)	4 (3–6)	0.117

*Data are shown in numbers (%) or IQR. MV, mechanical ventilation*.

**Table 2:** Platelet index before and after the ibuprofen treatment.

**Variates**	**PDA closure (*N* = 70)**	**PDA open (*N* = 59)**	***p***
PLT before the first course (× 10^9^/L)	212 (170–248)	183 (144–232)	0.024
PCT before the first course (%)	21.4 (16.9–23.4)	19.1 (14.8–23.9)	0.093
MPV before the first course (fl)	9.8 (9.3–10.9)	10.3 (9.4–10.9)	0.562
PDW before the first course (%)	16.9 (16.6–17.2)	17.0 (16.7–17.3)	0.644
Thrombocytopenia before the first course	12 (17.1%)	20 (33.9%)	0.028
PLT after the first course (× 10^9^/L)	265 (217–329)	216 (159–329)	0.091
PLT before the second course (× 10^9^/L)^#^	207 (155–313)	254 (196–348)	0.467
Thrombocytopenia before the second course^#^	2 (22.2%^#^)	3 (10%^#^)	0.572
PLT after the second course (× 10^9^/L)^#^	276 (204–361)	272 (208–345)	0.899

*PLT, platelet counts; PCT, plateletcrit; MPV, mean platelet volume; PDW, platelet distribution width; Thrombocytopenia, <150 × 10^9^/L;*

# *39 infants proceeded with second course of ibuprofen, 9 infants had their PDA closed, and 30 infants remained open*.

### ROC Estimation and Multivariable Regression Analysis

**Figure 2:**
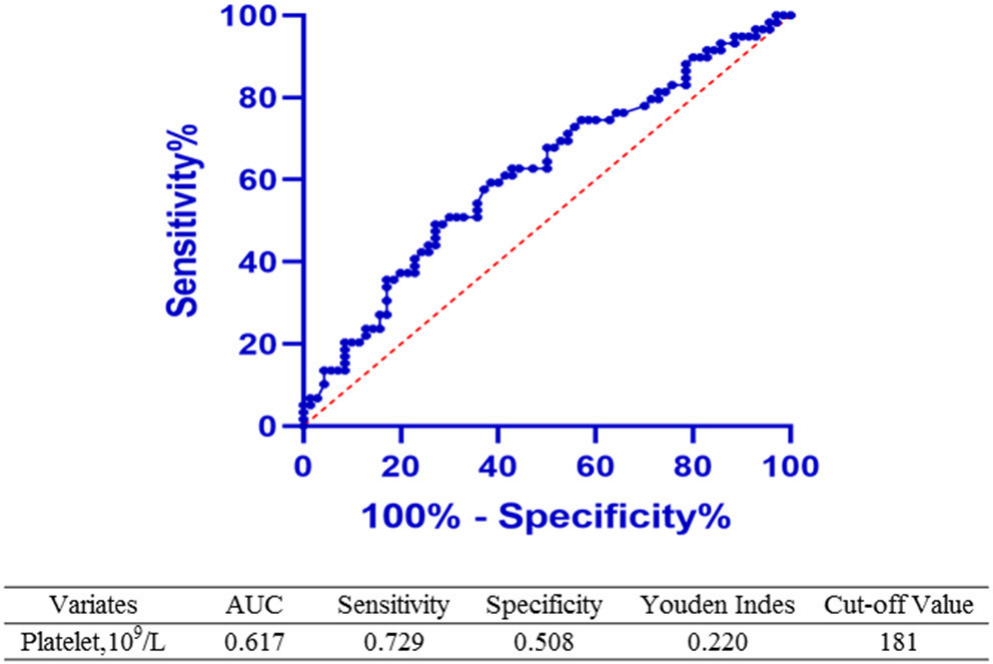
ROC curve and calculation of the cutoff value. A Receiver-operator curve was performed and the cutoff value of the platelet count before the initiation of treatment optimally predicting the closure of PDA was calculated. A platelet count ≥181 × 10^9^/L was concluded as the cutoff value with area under the curve (AUC, 0.617); the confidence interval (CI) was 0.483–0.685; sensitivity, 0.729; specificity, 0.508; and Youden index, 0.220.

ROC estimation was performed to determine the cutoff value of the platelet count before the initiation treatment for predicting the successful closure of hsPDA. A platelet count of 181 × 10^9^/L was calculated as the threshold with an area under the curve (AUC, 0.617); the confidence interval (CI) was 0.483–0.685; sensitivity, 0.729; specificity, 0.508; and Youden index, 0.220 (shown in Figure 2).

Multivariate logistic analysis was performed to assess the independent contribution of platelet level before ibuprofen treatment to the outcome of PDA closure (Table 3). A platelet count of ≥181 × 10^9^/L independently increased the closure rate of PDA after ibuprofen treatment (OR 2.556, 95% CI (1.101, 5.932), *p* = 0.029]. Besides, a higher gestational age showed a positive tendency of successful closure of PDA by ibuprofen while no statistical significance was reached (*p* = 0.062). Thrombocytopenia before the first course was not independently associated with hsPDA closure after ibuprofen treatment, although a tendency toward decreased chance of closure was observed (OR: 0.473, 95% CI: 0.190–1.178, *p* = 0.108).

**Table 3:** Multivariate regression analysis of successful closure of PDA by ibuprofen.

**Variates**	**OR**	**95% CI**	***p*-value**
Gestational age (weeks)	1.454	0.982–2.153	0.062
High PLT (× 10^9^/L)	2.556	1.101–5.932	0.029
Birth weight (g)	0.998	0.996–1.001	0.189
MV (>24 h)	0.651	0.281–1.509	0.317
Thrombocytopenia before the first course^#^	0.473^#^	0.190–1.178^#^	0.108^#^

*PLT, platelet counts; high PLT, PLT ≥181 × 10^9^/L; MV, mechanical ventilation;*

# *logistic regression was separately performed, adjusted for gestational age, birth weight and MV (>24 h)*.

## Discussion

In the current study, the rate of successful closure of hsPDA was 54.3% after the first ibuprofen course in VLBW infants, which is consistent with the data reported in the literature (1). Furthermore, a higher platelet count (≥181 × 10^9^/L) before ibuprofen treatment was found to be independently associated with the closure of hsPDA in VLBW infants without concurrent infection.

Since Echtler et al. (16) first reported that platelets are crucial for ductus arteriosus closure in mice and premature infants, several studies also analyzed the association between low platelet and the patency of ductus arteriosus in preterm infants, yielding conflicting findings (8, 17–20). Building up the complexity studies investigating whether platelet level affects the closure of PDA after pharmacotherapeutic agents also reported conflicting findings. Despite most of the studies reported no significant association between platelet level and closure of PDA after pharmacological therapy (6, 9, 21–23), a meta-analysis including 1,087 preterm infants showed that platelet level was significantly lower in infants with failed closure of PDA (11).

These conflicting findings drive us to reflect the possible explanations for those conflicting findings, which could be owing to the heterogeneity in the population (7, 10, 24), grouping on platelet level (21, 25), selected pharmacotherapeutic agent and dosage (6, 9, 10, 24, 26), study window (25), and other confounding factors. Platelet count and function are known to be dramatically affected by infection (27). Olsson et al. (28) reported that inflammatory status also influences the persistence of PDA probably by disturbing the function of platelets. Therefore, concurrent infection is a remarkable confounder, which affects both the cause (platelet) and the outcome (PDA closure). Although many researchers have tried to adjust this remarkable confounder in their statistical analysis (21, 29), we speculate that concurrent infection is such a dramatic and concealed confounder that may not be well-controlled by statistical models. Therefore, we excluded infants with this confounder by excluding infants with signs of infection 7 days before or after the ibuprofen treatment.

We found that a high level of platelet (≥181 × 10^9^/L) independently increased the probability of successful closure of hsPDA after ibuprofen treatment (OR: 2.556, 95% CI: 1.101–5.932, *p* = 0.029). The cutoff value was modestly higher than 150 × 10^9^/L, which is the diagnosis criteria of thrombocytopenia. Although thrombocytopenia was suggested to be related to ductus closure in preterm infants (30), other studies could not confirm these findings (21). Moreover, a randomized trial found maintaining a relatively high level of platelet by platelet transfusion in infants with thrombocytopenia did not facilitate the closure of DA (29). These controversies were nicely reviewed by Sallmon et al. (31) recently. In the current study, we also found that thrombocytopenia was not significantly associated with DA closure though a tendency was observed (OR: 0.473, 95% CI: 0.190–1.178). These conflicting results highlighted the need for both experimental and clinical studies to unravel the mechanism of DA closure and explore the potential interventions.

In the current study, we noticed that platelet count increased after each course of ibuprofen treatment (206 vs. 256 × 10^9^/L; 259 vs. 285 × 10^9^/L), irrespective of whether ductus was closed. In line with this finding, Sallmon et al. (9) also reported an increase in platelet count after the first course of cyclooxygenase inhibitor (COXI) cycle in both success and failed ductus closure. This could be partially attributed to the physiological increase of platelet count in the postnatal life of premature infants (32). Furthermore, ibuprofen might interfere with platelet function as the authors and other researchers suggested (9, 33). However, Sallmon et al. found a slight decrease in platelet count after the second and third round of COXI treatment, which was different from the current study. This might be owing to the heterogeneity of the study population.

In several studies, MPV, PDW, or platelet mass was reported to predict the persistence of PDA (17, 23, 34, 35), which is not confirmed in the current study. This could be explained by the fact that those platelet parameters are heavily influenced by an infection that frequently occurs to premature infants, whereas those infected infants were excluded from the current study. Besides, a delayed treatment also increases the failure of PDA closure; therefore, we only included infants who initiated the treatment within 7 days of age and observed no significant difference between infants with PDA closure and those with PDA open after ibuprofen treatment.

The main limitation of our study, apart from the retrospective nature, is the relatively small sample size due to the strict inclusion criteria. Besides, despite that, we included potential confounders influencing platelet level; other unknown factors related to platelet count and function may interfere with our results. Furthermore, physiological parameters of hsPDA such as oxygenation were not included in the analysis due to the frequent fluctuation in the early postnatal life.

In conclusion, a higher platelet count (≥181 × 10^9^/L) before ibuprofen treatment was independently associated with the successful closure of hsPDA.

## Data Availability Statement

The raw data supporting the conclusions of this article will be made available by the authors, without undue reservation.

## Ethics Statement

The studies involving human participants were reviewed and approved by the Shenzhen Maternity and Child Healthcare Hospital Institutional Ethical Committee (IEC). Written informed consent from the participants' legal guardian/next of kin was not required to participate in this study in accordance with the national legislation and the institutional requirements.

## Author Contributions

JZho and XC conceptualized and designed the study, and wrote the first draft of the manuscripts. JZho, BL, and YF carried out the clinical data collection. YY, JZha, and DZ performed data analysis. XC and CY reviewed and revised the manuscript. All authors have read and approved the final manuscript.

## Funding

This study was supported by the Guangdong Basic and Applied Science Committee (2020B1515120034 to CY), the Shenzhen Fund for Guangdong Provincial High-level Clinical Key Specialties (SZGSP009), and the Shenzhen Science and Technology Innovation Committee (JCYJ20190809170009528 to XC).

## Conflict of Interest

The authors declare that the research was conducted in the absence of any commercial or financial relationships that could be construed as a potential conflict of interest.

## Publisher's Note

All claims expressed in this article are solely those of the authors and do not necessarily represent those of their affiliated organizations, or those of the publisher, the editors and the reviewers. Any product that may be evaluated in this article, or claim that may be made by its manufacturer, is not guaranteed or endorsed by the publisher.
